# Data on the aquaporin gene expression differences among ρ^0^, clinically relevant radioresistant, and the parental cells of human cervical cancer and human tongue squamous cell carcinoma

**DOI:** 10.1016/j.dib.2018.08.025

**Published:** 2018-08-15

**Authors:** Yuko Takashi, Kazuo Tomita, Yoshikazu Kuwahara, Hideki Nabika, Kento Igarashi, Taisuke Nagasawa, Akihiro Kurimasa, Manabu Fukumoto, Yoshihiro Nishitani, Tomoaki Sato

**Affiliations:** a Department of Applied Pharmacology, Kagoshima University Graduate School of Medical and Dental Sciences, 8-35-1 Sakuragaoka, Kagoshima 890-8544, Japan; b Department of Restorative Dentistry and Endodontology, Kagoshima University Graduate School of Medical and Dental Sciences, 8-35-1 Sakuragaoka, Kagoshima 890-8544, Japan; c Division of Radiation Biology and Medicine, Faculty of Medicine, Tohoku Medical and Pharmaceutical University, 1-15-1, Fukumuro, Aoba, Miyagino, Sendai, Miyagi 983-8536, Japan; d Department of Material and Biological Chemistry, Faculty of Science, Yamagata University, 1-4-12 Kojirakawa, Yamagata 990-8560, Japan; e Department of Molecular Pathology, Tokyo Medical University, 6-1-1, Shinjuku, Shinjuku, Tokyo 160-0022, Japan

**Keywords:** AQP, aquaporin, CRR, clinically relevant radioresistant, HeLa, human cervical cancer, SAS, human tongue squamous cell carcinoma, mtDNA, mitochondrial DNA, nDNA, nuclear DNA, qPCR, quantitative PCR, FBS, Fetal Bovine Serum, Mitochondria, Aquaporin, Hydrogen peroxide, ρ^0^ cells, Clinically relevant radioresistant cells

## Abstract

We present data about mitochondrial DNA (mtDNA) copy number and aquaporin (AQP) gene expression in clinically radioresistant (CRR), ρ^0^, and their parental cells from human cervical cancer and human tongue squamous cell carcinoma. In both ρ^0^ and CRR cells, the mtDNA copy number was lower than for the parental strain. In addition, the obtained data suggest an association between the gene expression levels of AQP (1, 3, 8, and 9) and the difference in hydrogen peroxide (H_2_O_2_) sensitivity between ρ^0^ and CRR cells. Here, the composition of cell culture medium differs between CRR and ρ^0^ cells. To compare the gene expression of AQPs between ρ^0^ and CRR cells, therefore, we showed the data as the ratio to that in their parental cells.

**Specifications Table**

**Table:** 

Subject area	*Cancer science*
More specific subject area	*Cancer cell biology*
Type of data	*Table and figure*
How data were acquired	*Polymerase chain reaction*
Data format	*Raw and analyzed data*
Experimental factors	*Mitochondrial DNA copy number and aquaporin gene expression*
Experimental features	*Comparative analysis of aquaporin gene expression on CRR, ρ^0^, and their parental cells*
Data source location	*Kagoshima City, Japan*
Data accessibility	*Data are available with this article*.

**Value of the data**

• The data set is valuable for the scientific community that requires information regarding functional molecules of CRR cells.
• The data are suitable for comparing the properties between CRR and ρ^0^ cells.
• The data could promote further research about more effective methods of anti-cancer therapy.

## 1 Data

**Fig. 1:**
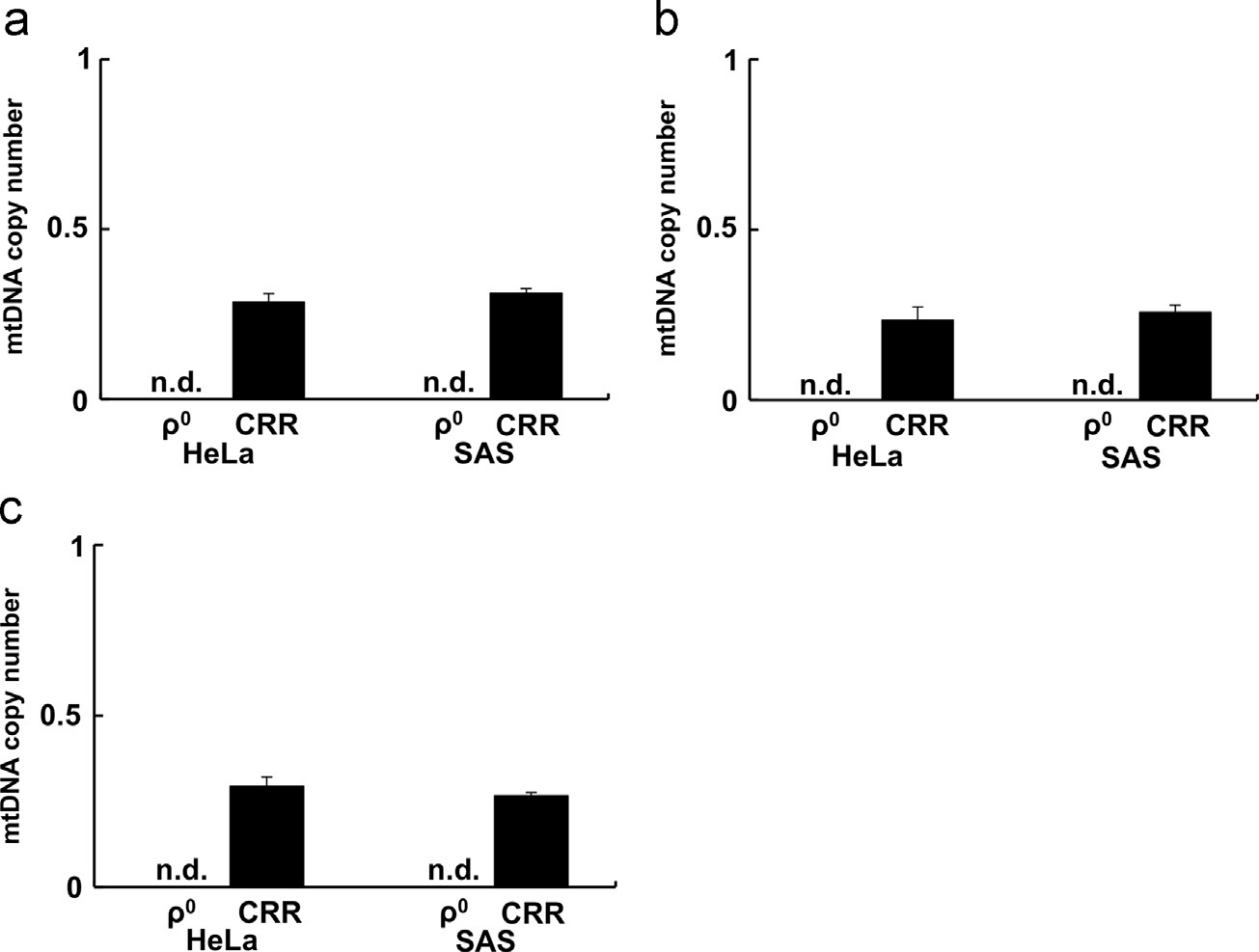
mtDNA copy numbers of ND2, COX2, and ATP6 in ρ^0^ and CRR cells of HeLa and SAS. The mtDNA copy numbers of CRR and ρ^0^ cells relative to those of their parents were measured by qPCR. For each cell line sample, three replicates were used to amplify mtDNA and nDNA. The mtDNA copy number is calculated by the ratio of mtDNA and nDNA (mtDNA/nDNA), and the copy number values on the *Y* axis in the figure are expressed as the ratio relative to that of each parental cell. (a) ND2, (b) COX2, and (c) ATP6. The mtDNA copy number of ρ^0^ cells was not detected and that of CRR cells was <0.5 in comparison with that of each parental cell. Each experiment was performed in triplicate. The results are presented as mean ± S.D. S.D. were calculated with the following the formula. S.D. = [(S.D._(Parent)_/mean_(Parent)_)^2^ + (S.D._(ρ0 or CRR)_/mean_(ρ0 or CRR)_)^2^]^1/2^ × (mean_(ρ0 or CRR)_/mean_(Parent)_).

In spite of mitochondrial hypofunction in ρ^0^ and CRR [1] cells, ρ^0^ and CRR cells have completely opposite patterns of sensitivity to H_2_O_2_ [2], [3]. AQP, a water channel, is essential for the permeation of small molecules such as H_2_O_2_ through the cell membrane. Therefore, the sensitivity to H_2_O_2_ may be due to a difference in AQP gene expression. The data presented here include mtDNA copy numbers and AQP gene expression comparing those of parental, ρ^0^, and CRR cells in human cervical cancer (HeLa) and human tongue squamous cell carcinoma (SAS). All data are shown as the ratio to the gene expression of parental cells. The ND2, COX2, and ATP6 genes are encoded by mtDNA and produce components of the mitochondrial electron transport chain complex I, IV, and V proteins, respectively. To compare the mitochondrial function, the mtDNA copy numbers were investigated in the ρ^0^ and CRR cells of HeLa and SAS (Fig. 1, Table 1).

**Table 1:** mtDNA copy number of parent, CRR, and ρ^0^ cells.

	**ND2**	**COX2**	**ATP6**
HeLa-Parent	152 ± 12	182 ± 15	222 ± 16
HeLa-CRR	43 ± 1	42 ± 6	65 ± 4
SAS-Parent	90 ± 3	116 ± 5	162 ± 6
SAS-CRR	28 ± 0	30 ± 2	43 ± 2
HeLa-Parent	53 ± 8	63 ± 8	78 ± 22
HeLa-ρ^0^	0 ± 0	0 ± 0	0 ± 0
SAS-Parent	60 ± 15	72 ± 25	103 ± 45
SAS-ρ^0^	0 ± 0	0 ± 0	0 ± 0

The data are raw data of Fig. 1. The ratio of mtDNA and nDNA (mtDNA/nDNA) in genomic DNA is shown as the mean ± S.D.

**Fig. 2:**
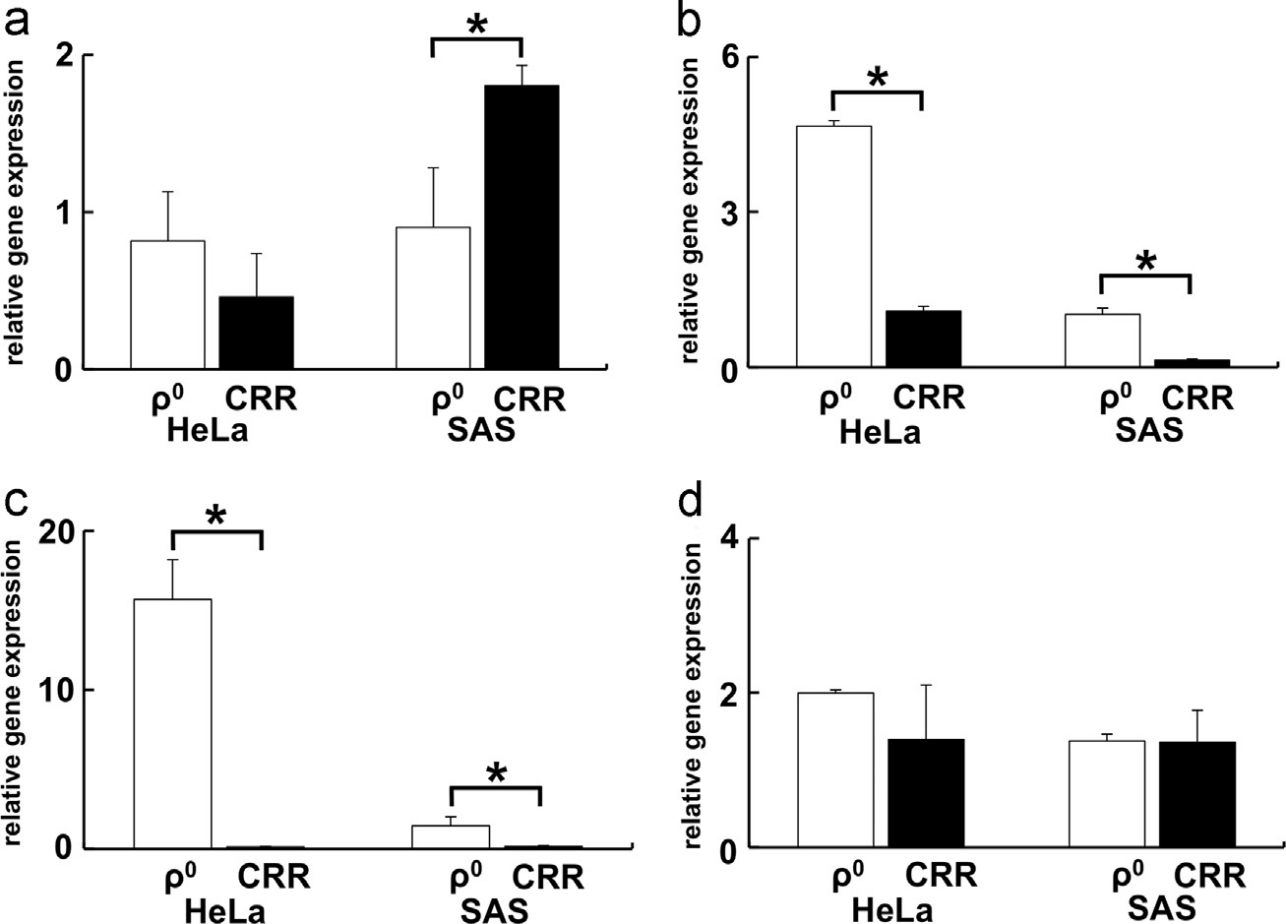
Gene expression of the AQPs having H_**2**_O_**2**_ permeability in ρ^0^ and CRR cells. The AQP gene expression values are expressed as the ratio relative to that of each parental cell. (a) AQP1, (b) AQP3, (c) AQP8, and (d) AQP9. In CRR cells, the expression of AQP3 and 8 genes was decreased in comparison with that in ρ^0^ cells. There was no common tendency regarding a decrease of gene expression between HeLa and SAS in AQP1 and 9. Each experiment was performed in triplicate. The results are presented as mean ± S.D. S.D. were calculated as in Fig. 1. *: *p* < 0.05 by Student׳s *t*-test.

**Fig. 3:**
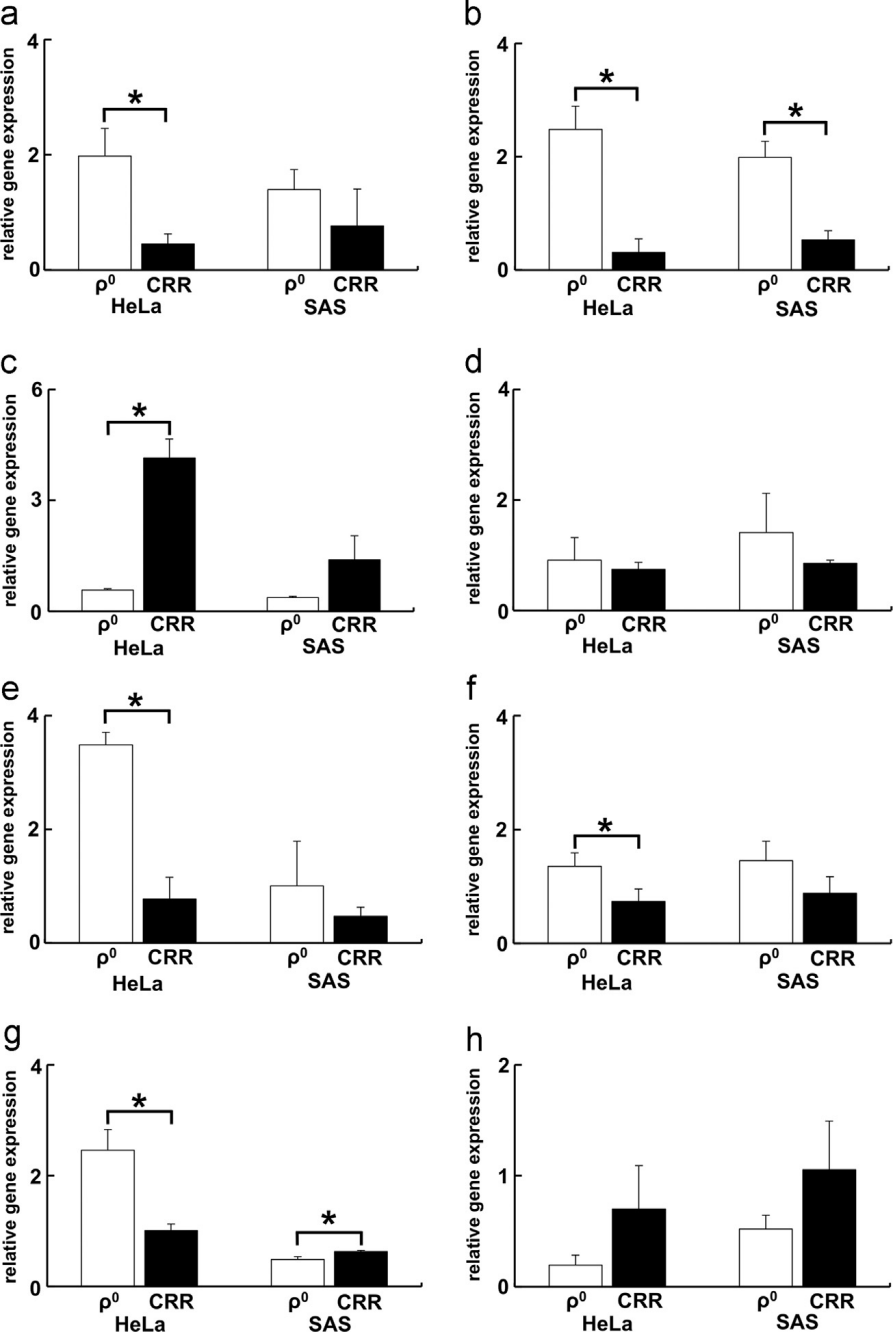
Gene expression of the other AQPs (AQP 0, 2, 4, 5, 6, 7, 10, 11, and 12) in ρ^0^ and CRR cells. (a) AQP0, (b) AQP2, (c) AQP5, (d) AQP6, (e) AQP7, (f) AQP10, (g) AQP11, and (h) AQP12. AQP4 gene expression was not detected in ρ^0^ and CRR cells (data not shown). There were tendencies for decreases of AQP0, 2, 6, 7, and 10 gene expression in the CRR cells compared with the level in ρ^0^, but the gene expression of AQP5 and 12 in CRR cells was increased compared with that in ρ^0^ cells. There was no common tendency regarding the gene expression of AQP11 in ρ^0^ and CRR cells. Each experiment was performed in triplicate. The results are presented as mean ± S.D. S.D. were calculated as in Fig. 1. *: *p* < 0.05 by Student׳s *t*-test.

Reportedly, H_2_O_2_ can permeate the plasma membrane via AQP1, 3, 8, and 9 [4], [5], [6], [7]; therefore, the gene expression of AQPs was investigated (Fig. 2). The other type of AQP gene expression is shown in Fig. 3. Furthermore, because all water-permeable AQPs are suggested to be permeable to H_2_O_2_ [5], we compared the gene expression of AQP1, 3, 8, and 9 and all AQPs in ρ^0^ and CRR cells with that of their parental cells (Table 2, Table 3).

**Table 2:** Gene expression level of each AQP. The data are raw data of Fig. 2, Fig. 3. All data are normalized to an internal standard (β-actin) and show the mean ± S.D.

	AQP0	AQP1	AQP2	AQP3
HeLa-Parent	6.38 × 10^−5^ ± 2.12 × 10^−5^	1.59 × 10^−5^ ± 6.41 × 10^−6^	3.10 × 10^−6^ ± 2.60 × 10^−7^	5.76 × 10^−5^ ± 3.98 × 10^−6^
HeLa-CRR	2.89 × 10^−5^ ± 5.56 × 10^−6^	7.33 × 10^−6^ ± 3.28 × 10^−6^	9.66 × 10^−7^ ± 7.34 × 10^−7^	6.23 × 10^−5^ ± 3.73 × 10^−6^
SAS-Parent	8.98 × 10^−6^ ± 4.46 × 10^−6^	3.29 × 10^−6^ ± 9.22 × 10^−8^	9.97 × 10^−7^ ± 1.08 × 10^−7^	5.25 × 10^−6^ ± 1.46 × 10^−7^
SAS-CRR	6.88 × 10^−6^ ± 4.61 × 10^−6^	5.93 × 10^−6^ ± 3.90 × 10^−7^	5.34 × 10^−7^ ± 1.49 × 10^−7^	7.46 × 10^−7^ ± 1.36 × 10^−7^
	AQP5	AQP6	AQP7	AQP8
HeLa-Parent	7.92 × 10^−6^ ± 3.39 × 10^−7^	1.50 × 10^−5^ ± 1.45 × 10^−7^	1.92 × 10^−5^ ± 6.56 × 10^−6^	5.24 × 10^−5^ ± 2.13 × 10^−6^
HeLa-CRR	3.29 × 10^−5^ ± 3.78 × 10^−6^	1.12 × 10^−5^ ± 1.90 × 10^−6^	1.50 × 10^−5^ ± 5.19 × 10^−6^	6.54 × 10^−6^ ± 1.20 × 10^−6^
SAS-Parent	3.58 × 10^−6^ ± 1.65 × 10^−6^	1.24 × 10^−5^ ± 6.33 × 10^−7^	6.95 × 10^−6^ ± 1.16 × 10^−6^	3.75 × 10^−5^ ± 9.55 × 10^−6^
SAS-CRR	5.00 × 10^−6^ ± 2.96 × 10^−7^	1.05 × 10^−5^ ± 5.03 × 10^−7^	3.28 × 10^−6^ ± 9.68 × 10^−7^	5.48 × 10^−6^ ± 2.29 × 10^−6^
	AQP9	AQP10	AQP11	AQP12
HeLa-Parent	5.46 × 10^−6^ ± 2.70 × 10^−6^	1.38 × 10^−5^ ± 3.43 × 10^−6^	1.60 × 10^−4^ ± 3.09 × 10^−6^	1.78 × 10^−5^ ± 3.98 × 10^−6^
HeLa-CRR	7.62 × 10^−6^ ± 7.91 × 10^−7^	1.02 × 10^−5^ ± 1.58 × 10^−6^	1.62 × 10^−4^ ± 1.87 × 10^−5^	1.25 × 10^−5^ ± 6.34 × 10^−6^
SAS-Parent	6.22 × 10^−6^ ± 1.54 × 10^−6^	5.25 × 10^−6^ ± 1.61 × 10^−6^	5.21 × 10^−4^ ± 7.66 × 10^−6^	1.64 × 10^−5^ ± 6.22 × 10^−6^
SAS-CRR	8.47 × 10^−6^ ± 1.47 × 10^−6^	4.61 × 10^−6^ ± 5.79 × 10^−7^	3.28 × 10^−4^ ± 8.66 × 10^−6^	1.73 × 10^−5^ ± 3.00 × 10^−6^
	AQP0	AQP1	AQP2	AQP3
HeLa-Parent	1.71 × 10^−5^ ± 4.07 × 10^−6^	1.46 × 10^−5^ ± 4.66 × 10^−6^	3.74 × 10^−6^ ± 3.80 × 10^−7^	6.06 × 10^−5^ ± 1.10 × 10^−6^
HeLa-ρ^0^	3.39 × 10^−5^ ± 1.44 × 10^−6^	1.19 × 10^−5^ ± 2.62 × 10^−6^	9.28 × 10^−6^ ± 1.22 × 10^−6^	2.82 × 10^−4^ ± 3.54 × 10^−6^
SAS-Parent	1.90 × 10^−5^ ± 3.64 × 10^−6^	1.02 × 10^−5^ ± 1.53 × 10^−6^	4.14 × 10^−6^ ± 3.66 × 10^−7^	3.15 × 10^−5^ ± 8.34 × 10^−7^
SAS-ρ^0^	2.66 × 10^−5^ ± 4.16 × 10^−6^	9.22 × 10^−6^ ± 3.62 × 10^−6^	8.23 × 10^−6^ ± 9.11 × 10^−7^	3.21 × 10^−5^ ± 3.88 × 10^−6^
	AQP5	AQP6	AQP7	AQP8
HeLa-Parent	2.26 × 10^−5^ ± 1.44 × 10^−6^	1.03 × 10^−5^ ± 3.34 × 10^−6^	1.77 × 10^−5^ ± 5.31 × 10^−7^	3.45 × 10^−6^ ± 4.33 × 10^−7^
HeLa-ρ^0^	1.30 × 10^−5^ ± 3.58 × 10^−7^	9.38 × 10^−6^ ± 2.84 × 10^−6^	6.17 × 10^−5^ ± 3.45 × 10^−6^	5.40 × 10^−5^ ± 5.40 × 10^−6^
SAS-Parent	7.82 × 10^−6^ ± 5.32 × 10^−7^	7.04 × 10^−6^ ± 1.73 × 10^−6^	1.01 × 10^−5^ ± 2.63 × 10^−6^	2.48 × 10^−5^ ± 3.93 × 10^−6^
SAS-ρ^0^	2.95 × 10^−6^ ± 8.31 × 10^−8^	9.92 × 10^−6^ ± 4.38 × 10^−6^	1.01 × 10^−5^ ± 7.42 × 10^−6^	3.54 × 10^−5^ ± 1.35 × 10^−5^
	AQP9	AQP10	AQP11	AQP12
HeLa-Parent	2.68 × 10^−4^ ± 5.14 × 10^−6^	7.17 × 10^−6^ ± 5.82 × 10^−7^	1.04 × 10^−4^ ± 1.52 × 10^−5^	3.32 × 10^−5^ ± 5.57 × 10^−6^
HeLa-ρ^0^	5.35 × 10^−4^ ± 6.37 × 10^−6^	9.69 × 10^−6^ ± 1.53 × 10^−6^	2.55 × 10^−4^ ± 1.13 × 10^−5^	6.48 × 10^−6^ ± 2.85 × 10^−6^
SAS-Parent	3.50 × 10^−5^ ± 1.02 × 10^−6^	1.11 × 10^−5^ ± 3.00 × 10^−7^	5.96 × 10^−4^ ± 2.81 × 10^−5^	9.28 × 10^−5^ ± 2.09 × 10^−5^
SAS-ρ^0^	4.80 × 10^−5^ ± 2.90 × 10^−6^	1.61 × 10^−5^ ± 3.80 × 10^−6^	2.90 × 10^−4^ ± 2.56 × 10^−5^	4.82 × 10^−5^ ± 4.45 × 10^−6^

**Table 3:** Total gene expression of AQP0–12 and of AQP1, 3, 8, and 9 in comparison with that in each parental cell.

		**CRR**	**ρ**^**0**^
AQP Gene expression (AQP0-12)	HeLa	0.83	2.28
	SAS	0.63	0.63
AQP Gene expression (AQP1, 3, 8, 9)	HeLa	0.64	2.55
	SAS	0.39	1.23

The gene expression of AQP was calculated as the sum of each AQP gene expression, and the values are expressed as gene expression ratio values of CRR and ρ^0^ cells relative to those of their parental cells.

## 2 Experimental design, materials, and methods

### 2.1 Cell lines

HeLa and SAS cell lines were obtained from the Cell Resource Center for Biomedical Research, Institute of Development, Aging and Cancer, Tohoku University (Sendai, Japan).

### 2.2 ρ^0^ cells

It is reported that ρ^0^ cells do not have mtDNA [8]. Here, ρ^0^ cells were established by culturing in RPMI1640 containing 5% FBS, 50 ng/mL EtBr, 50 μg/mL uridine, and 110 μg/mL sodium pyruvate for 3–4 weeks [9].

### 2.3 CRR cells

The establishment of CRR cell lines was conducted by stepwise increase of the X-ray dose of fractionated radiation from 0.5 to 2 Gy/day *in vitro* [1].

### 2.4 Measurement of mtDNA copy numbers in ρ^0^ and CRR cells

mtDNA copy numbers in parental, ρ^0^, and CRR cells were measured in accordance with the procedure described in our previous report [3]. Genomic DNA was extracted by phenol extraction [10]. Ten nanograms of DNA was used for quantitative PCR (qPCR) to detect mtDNA (ND2, COX2, and ATP6) and nuclear DNA (nDNA; β-actin).

The qPCR reactions were performed with Step One Plus (Applied Biosystems, Foster City, CA, USA) using the THUNDERBIRD^®^ SYBR qPCR Mix (TOYOBO Co. Ltd., Osaka, Japan). Following qPCR, the ratio of mtDNA/nDNA was calculated. Each experiment was performed in triplicate. Primer sequences are listed in Table 4, Table 5. The conditions of qPCR are shown in Table 6.

**Table 4:** The primer sequences of ND2, COX2, and ATP6 in this study.

**Primer name**	**Primer sequence**
ND2-F	5′-GAAACAAGCTAACATGACTAACACCCTTAA-3′
ND2-R	5′-TATGATGGTGGGGATGATGAGGCTAT-3′
COX2-F	5′-TGAGCTGTCCCCACATTAGGCTTA-3′
COX2-R	5′-GGGCATGAAACTGTGGTTTGCTCC-3′
ATP6-F	5′-CACCTACACCCCTTATCCCCATAC-3′
ATP6-R	5′-GGTAGAGGCTTACTAGAAGTGTGA-3′

**Table 5:** Primer sequences for AQP0–12 and β-actin in this study.

**Primer name**	**Primer sequence**
AQP0-F	5′-GCAGCCTCCTGTACGACTTTCTTCTCTT-3′
AQP0-R	5′-GGCCTGGGTGTTCAGTTCAACAGGTT-3′
AQP1-F	5′-TGGATTTTCTGGGTGGGGCCATTCAT-3′
AQP1-R	5′-TTCATCTCCACCCTGGAGTTGATGTC-3′
AQP2-F	5′-CTGGTACAGGCTCTGGGCCACATAA-3′
AQP2-R	5′-ATGTCTGCTGGCGTGATCTCATGGAG-3′
AQP3-F	5′-TTTTTACAGCCCTTGCGGGCTGGG-3′
AQP3-R	5′-ATCATCAGCTGGTACACGAAGACACC-3′
AQP4-F	5′-GGTGGCCTTTATGAGTATGTCTTCTGTC-3′
AQP4-R	5′-TTTTAGAATCAGGTCATCCGTCTCTACCTG-3′
AQP5-F	5′-TGCGGTGGTCATGAATCGGTTCAGC-3′
AQP5-R	5′-ACGCTCACTCAGGCTCAGGGAGTT-3′
AQP6-F	5′-TGGGAAGTTCACAGTCCACTGGGTC-3′
AQP6-R	5′-TCTACGGTGCCTGTGAGGATAGCC-3′
AQP7-F	5′-ACGGACCAGGAGAACAAC-3′
AQP7-R	5′-CCCAACCAGCAATGAAGG-3′
AQP8-F	5′-AACCACTGGAACTTCCACTGGATCTACT-3′
AQP8-R	5′-ATCTCCAATGAAGCACCTAATGAGCAGTC-3′
AQP9-F	5′-CTGTCATTGGAGGCCTCATCTATGTTCTT-3′
AQP9-R	5′-GTTCTGTCTTAAAGACTGAGTCAGGCTCT-3′
AQP10-F	5′-GAAGTCTTCAGTGCTGGTAATGGCTG-3′
AQP10-R	5′-CTTTGTGTTGAGCAGACACCAGATCCT-3′
AQP11-F	5′-AATCCAGCTTTGGCACTTTCGCTACATTTC-3′
AQP11-R	5′-TGCAGCCATGGAAGGAAAAAGCTGAACAT-3′
AQP12-F	5′-TTCTACGGCCAGAAGAACAAGTACCGA-3′
AQP12-R	5′-TCAGCTGGAATGTGGCCCCTCAAC-3′
β-actin-F	5′-AGAGCTACGAGCTGCCTGAC-3′
β-actin-R	5′-AGCACTGTGTTGGCGTACAG-3′

**Table 6:** Conditions for qPCR in this study. AQP4 gene expression was not detected in parental, ρ^0^, and CRR cells of HeLa and SAS. Therefore, the PCR condition of AQP4 is shown as “-.”.

Gene	PCR condition	A	95 °C 10 s, 60 °C 1 min
ND2	A	B	95 °C 10 s, 52.5 °C 10 s, 72 °C 30 s
COX2	A	C	95 °C 10 s, 55 °C 10 s, 72 °C 30 s
ATP6	A	D	95 °C 10 s, 60 °C 10 s, 72 °C 30 s
AQP	PCR condition		
0	D		
1	A		
2	C		
3	A		
4	–		
5	A		
6	B		
7	D		
8	A		
9	A		
10	D		
11	A		
12	D		

### 2.5 Gene expression of AQPs

The qPCR of AQP was conducted as described in a previous study [2], with slight modifications. All cDNAs were prepared by reverse transcription using ReverTra Ace (TOYOBO). Equivalent to 1 ng of total RNA was used for qPCR. Each experiment was performed in triplicate. Primer sequences of AQPs and β-actin are listed in Table 5. The conditions of qPCR are shown in Table 6.

### 2.6 Statistical analysis

Statistical analyses were performed using Student׳s *t*-test. *P*-values <0.05 were considered statistically significant.
