# Supplementary material for: Data on the aquaporin gene expression differences among ρ0, clinically relevant radioresistant, and the parental cells of human cervical cancer and human tongue squamous cell carcinoma
Source: Data Brief. 2018 Aug 15;20:402–10. doi: 10.1016/j.dib.2018.08.025 (PMC6116339; doi:10.1016/j.dib.2018.08.025)
Supplement: Supplementary file 1 — Supplementary material [file mmc1.docx]

Conflicts of Interests

Data on the aquaporin gene expression differences among ρ^0^, clinically relevant radioresistant, and the parental cells of human cervical cancer and human tongue squamous cell carcinoma

**by Yuko Takashi, Kazuo Tomita, Yoshikazu Kuwahara, Hideki Nabika, Kento Igarashi, Taisuke Nagasawa, Akihiko Kurimasa, Manabu Fukumoto, Yoshihiro Nishitani, Tomoaki Sato**

All authors declare that they have no conflicts of interest regarding the contents of this article.
